# Separating the effects of temperature and carbon allocation on the diel pattern of soil respiration in the different phenological stages in dry grasslands

**DOI:** 10.1371/journal.pone.0223247

**Published:** 2019-10-17

**Authors:** János Balogh, Szilvia Fóti, Marianna Papp, Krisztina Pintér, Zoltán Nagy

**Affiliations:** 1 Institute of Botany and Ecophysiology, Szent István University, Gödöllő, Hungary; 2 MTA-SZIE Agroecology Research Group, Szent István University, Gödöllő, Hungary; Universidade Federal de Santa Maria, BRAZIL

## Abstract

Diel variability of soil respiration is influenced by several factors including temperature and carbon allocation as the most significant ones, co-varying on multiple time scales. In an attempt to disentangle their effects we analyzed the dynamics of soil respiration components using data from a three-year soil respiration study. We measured CO_2_ efflux in intact, root-excluded and root- and mycorrhizal fungi excluded plots and analyzed the diel variability in different phenological stages. We used sine wave models to describe the diel pattern of soil respiration and to disentangle the effects of temperature from belowground carbon allocation based on the differences between component dynamics inferred from the fitted models. Rhizospheric respiration peaked 8–12 hours after GPP peak, while mycorrhizal fungi respiration had a longer time lag of 13–20 hours. Results of δ^13^CO_2_ isotopic signals from the respiration components showed similar patterns. It was found that drought affected the component respiration rates differently. Also, the speed and the amount of carbon allocation to the roots as well as to the mycorrhizal fungi was reduced under drought. We conclude that the diel variability of soil respiration is the result of the integrated patterns of temperature- and carbon allocation-driven components in dry grasslands and their share depends on their phenological stages and stress state.

## Introduction

The carbon balance of ecosystems is the sum of sink and source activities and exhibit large seasonal and interannual variability [1]. Since the major part of the source activity is the result of soil respiration (R_s_), the variability of this CO_2_ flux has a significant relevance in the carbon balance [2]. Soil respiration is a highly complex process including a wide range of soil biota (autotrophic and heterotrophic functioning) and different pathways of carbon cycling (decomposition, carbon allocation), all being under the control of environmental and biotic drivers [3–5]. Improving our understanding of the links between these processes and their drivers on multiple time scales has a major importance in decreasing uncertainties concerning the carbon cycle models of ecosystems [6] as well as in decreasing uncertainties related to ecosystem respiration estimations [7].

Drought-prone ecosystems are key members of the global carbon cycling contributing significantly to the interannual variability of the global CO_2_ sink [8]. Their carbon balance is strongly associated with the variations in precipitation and temperature and can turn from carbon sink to carbon source due to drought [9,10]. Dry grasslands, often subjected to drought, experience strong seasonality and their functioning depends upon the presence and activity of the canopy [11,12]. Therefore, the coupling between aboveground gross primary productivity (GPP) and carbon allocation to roots and root-associated organisms varies depending on the season [13]. The vegetation type defines the phenological stage and the range of productivity on a seasonal scale, as well as the coupling of photosynthesis and belowground respiration on the diel scale [3,14]. Seasonality induces changes in belowground carbon allocation patterns with the amounts of carbon allocated to the roots and to the mycorrhizal fungi partners being highly variable in the different seasons [15,16] and the use of fresh assimilates for respiration or storage also depending on the phenological stage [17].

The major factor hampering the quantification of the carbon allocation driven part of soil respiration is the temperature co-varying with GPP on seasonal and diel time scales [13,18,19]. The sole effect of temperature on R_s_ was extensively studied [20,21] and the correlation was used as a basis for soil respiration models but potential artefacts were already highlighted as well [7,22]. The observed time lag between soil temperature and R_s_ and the diel variation of R_s_ was often attributed to the depth at which temperature was measured [23,24] or to the gas transport properties of the soil [25] combined with other factors [3,26]. Furthermore, attempts were also made to explain the time lags in the different responses of soil respiration components. However, changes in R_s_ can even precede those occurring in soil temperature especially in dry ecosystems [27,28].

Generally, the autotrophic component of R_s_ is determined by the amount of CO_2_ produced by plant roots and associated microorganisms (rhizospheric microbes, mycorrhizal fungi), while heterotrophic respiration is represented by the amount of CO_2_ produced by microbial decomposition of SOM [29]. R_s_ partitioning could be useful for estimating the contribution of the different components and for revealing the potential effects of the drivers on the autotrophic and heterotrophic components of the soil. Heterotrophic activities were found to be more sensitive to temperature than the autotrophic ones [19,30], while less sensitive to drought conditions [31], although the responses can vary with the type of vegetation [32]. However, both autotrophic and heterotrophic components receive assimilates from the shoots [33,34], therefore the dynamics of belowground carbon allocation should also be included in the analysis of component responses.

Our objective was to characterize the diel patterns of the temperature and carbon allocation driven parts of soil respiration and their changes in the different phenological stages. In our attempt to do so, we analyzed the diel variability of soil respiration as well as the CO_2_ efflux of root-excluded and root- and mycorrhizal fungi-excluded plots in three consecutive years. Measurements of δ^13^C were also conducted together with the CO_2_ efflux measurements in the growing season in one of the study years. Significant drought periods intervened in the growing seasons in two of the studied years allowing us to analyze the effect of drought on the diel pattern of soil CO_2_ efflux.

Our analysis focused on the (1) differences in time lags between GPP and the respiration components; and the (2) share of the carbon allocation driven part in soil respiration in the different phenological stages. We hypothesize that it is the diel dynamics of carbon allocation that drive a significant part of soil respiration in the growing season and it is influenced by the drought periods resulting in seasonally different diel patterns. We also hypothesize that the time lag between GPP and respiration is longer in mycorrhizal fungi than in roots and rhizosphere due to the allocation patterns within the soil.

## Methods

### Site description

The vegetation at the Bugac site (46.69° N, 19.6° E, 114 m above sea level) is a dry sandy grassland dominated by *Festuca pseudovina*, *Carex stenophylla* and *Cynodon dactylon* and it has been under extensive management (grazing) for the last 20 years [35]. The ten-year mean annual precipitation (2004–2013) was 575 mm and the mean annual temperature reached 10.4 °C. According to the FAO classification [36] the soil type is Chernozem with a relatively high organic carbon content, the soil texture is a sandy loam with a sand:silt:clay ratio of 81:11:8% in the topsoil layer [37].

### Measured and calculated CO_2_ efflux components

In September 2010 ten soil cores (160 mm in diameter and 800 mm in depth, except No. 9, which had 500 mm diameter) were drilled and the roots have been removed by sieving. During the drilling 4 soil layers were separated: 0–10 cm, 10–30 cm, 30–50 cm and 50–80 cm The root-free soil was packed layer by layer into PVC tubes. Five tubes were used to exclude both roots and mycorrhiza. Walls of another 5 tubes were partially removed and replaced by inox meshes (40 μm pore size) to exclude roots, while ensuring that the mycorrhiza filaments can grow into the tubes [38]. These root- and mycorrhiza free and only root-free soil cores were placed at a distance of 6 m from the eddy covariance tower to South (S1 Fig). The distance between the soil cores/tubes was 50 cm.

Soil CO_2_ efflux and its isotopic signal were measured in plots of:

- undisturbed soil: soil respiration, R_s_,
- root-excluded soil = without roots but with arbuscular mycorrhizal fungi, R_basal+myc_.
- soil without roots and arbuscular mycorrhizal fungi = basal respiration, R_basal_,

The presence of mycorrhizal fungi filaments in R_basal+myc_ and R_s_ plots was confirmed by microbiological investigations as well as overall soil enzymatic activity measured in all plots [39].

Although it was not possible to calculate the respiration rates of the autotrophic and heterotrophic components directly, we used the differences between the average respiration rates of specific plots for describing the diel pattern of the components of soil respiration. According to our approach we calculated the respiration components as follows:

- mycorrhizospheric component [29]:
  $$R_{myc+rhizo}=R_{s}-R_{basal}$$ (1)
- mycorrhizal fungi component:
  $$R_{myc}=R_{basal+myc}-R_{basal}$$ (2)
- rhizospheric component:
  $$R_{rhizo}=R_{s}-R_{basal+myc}$$ (3)

Table 1 contains the description of measured and calculated soil respiration components and abbreviations used in the study following the terminology of Moyano et al. [29]. Priming effect was not partitioned, therefore it was included in the respective R_myc+rhizo_, R_myc_ and R_rhizo_ components.

**Table 1:** Measured and calculated components of soil respiration used in this study.

	Abbreviation	Description	Priming included
Measured	R_s_	soil respiration, containing all of the components, measured in undisturbed soil	yes
	R_basal+myc_	basal and mycorrhizal fungi respiration, measured in root-excluded soil	partly
	R_basal_	basal respiration, respiration of SOM decomposition without the priming effect	no
Calculated	R_myc+rhizo_	respiration of roots, rhizosphere microorganisms and mycorrhizal fungi	yes
	R_rhizo_	respiration of roots and rhizosphere microorganisms	partly
	R_myc_	respiration of mycorrhizal fungi filaments	partly

### Gas exchange measuring systems

Different gas exchange systems were used in the present study: eddy-covariance system (EC), automated soil respiration measuring system (SRS) and an isotopic CO_2_ analyzer (cavity ring-down spectroscopy, CRDS-technique) was connected to the SRS in 2013 (see Isotopic measurements). The size of the EC flux footprint area was larger by several orders of magnitude than the area covered by the SRS. Care was taken during the establishment of the experiment to install the partitioning set-up with the same average soil characteristics and vegetation composition and cover as found in the EC footprint area [35]. Hence, the NEE and GPP estimates obtained in this way can be considered to be representative also of the small-scale SRS and isotope measurements.

Data from 6^th^ July 2011 to 12^th^ May 2014 were analyzed in this study.

### Eddy-covariance setup

The EC system at the Bugac site has been measuring the CO_2_ and sensible and latent heat fluxes continuously since 2002. In dry years the grassland can turn into a net carbon source [9], but the longer-term annual sums of net ecosystem exchange (NEE) show it to be a net sink, ranging from –171 to +106 g C m^–2^ yr^–1^ [40,41] with a -100 g C m^–2^ yr^–1^ average.

The EC system consists of a CSAT3 sonic anemometer (Campbell Scientific, USA) and a Li-7500 (Licor Inc, USA) open-path infra-red gas analyzer (at the height of 4 m, anemometer direction: north), both connected to a CR5000 data logger (Campbell Scientific, USA) via an SDM (synchronous device for measurement) interface. Additional measurements used in the present study included air temperature and relative humidity (HMP35AC, Vaisala, Finland), precipitation (ARG 100 rain gauge, Campbell, UK), global radiation (dual pyranometer, Schenk, Austria from 2002 and CMP3, Kipp&Zonen, The Netherlands from 2013) incoming and reflected photosynthetically active radiation (SKP215, Campbell, UK), volumetric soil moisture content (CS616, Campbell, UK) and soil temperature (105T, Campbell, UK). Surface temperature (T_surf_) was measured by 3 cm above the ground under the leaves. These measurements were performed as described by Nagy et al. and Pintér et al. [9,40]. Fluxes of sensible and latent heat and CO_2_ were processed by EddyPro^®^ [42] using double rotation, linear detrending and WPL correction [43]. Gap-filling and flux partitioning was performed by the REddyProc online data processing tool [44].

### Soil respiration system

Automated soil respiration system consisting of ten chambers was set up in July 2011. The system is an open dynamic one, consisting of an SBA-4 infrared gas analyzer (PPSystems, UK), pumps, flow meters (D6F-01A1-110, Omron Co., Japan), electro-magnetic valves, and PVC/metal soil chambers. The chambers were 10.4 cm high with a diameter of 5 cm, covering a soil surface area of 19.6 cm^2^. The flow rate through the chambers was 300 ml min^-1^, exchanging the air in the chamber in 40 seconds. The PVC chambers were enclosed in a white metal cylinder with 2 mm airspace in between to stabilize the chamber and to prevent warming by direct radiation. Four vent holes with a total area of 0.95 cm^2^ were drilled in the top of the chambers. Vent holes also served to allow precipitation to drip into the chambers. The system causes minor disturbances in the soil structure and the spatial structure of the vegetation. It can be applied without cutting the leaves/shoots of the plants, so it does not disturb the transport processes taking place within the plant stems and roots. It is suitable for continuous and long-term unattended measurements of soil CO_2_ efflux and was used in previous experiments [45]. The soil respiration chambers contained no standing aboveground plant material.

Measurements with the SRS chambers were carried out as follows: R_s_ was measured by 6 SRS chambers within the area in random positions (cf. S1 Fig), R_basal+myc_ was measured by 2 SRS chambers in plots of PVC tubes with inox mesh and R_basal_ was measured by 2 SRS chambers in intact PVC tubes. Positions of the chambers were changed every 2 weeks within the corresponding treatments during the study period that is among the 10 tubes (R_basal+myc_, R_basal_) or among randomly selected locations within the undisturbed area (R_s_) to obtain sequential spatial replications for each treatment type. The system operated in sequential mode during the whole study period: it was idle after one hour of operation, during which the chambers were measured twice (two cycles of measurements). Despite the vent holes in the chambers there was a build-up of CO_2_ concentration within the chambers before the system was idle, therefore the data recorded in the first 30 minutes (first cycle) was excluded from the analysis. One measurement of each chamber lasted 3 minutes with the reference/analysis air being switched in every 7 seconds. This procedure resulted in 12 measurements (36 min)/day for each chamber.

Two soil moisture and soil temperature sensors (5TM, Decagon Devices, USA) were also attached to the system measuring soil temperature and moisture of an R_s_ plot (T_Rs_) and an R_basal_ plot (T_Rbasal_) at a depth of 5–9 cm.

### Isotopic (^13^CO_2_) measurements

A Picarro G1101-i gas analyzer (Picarro Inc., CA, USA) was attached to the soil respiration system from 15^th^ May to 12^th^ November in 2013. Since the CRDS had much slower response than the SRS, every second chamber of the SRS was measured by the CRDS. Out of these five chambers, 3 chambers measured R_s_, one of them measured R_het+myc_ and one of them measured R_het_. Regularly changing the position of the chambers ensured the spatial replications of the measurements [31].

The CRDS system measured the isotopic composition of the reference air (in the grass canopy 10 cm above the surface) when the soil respiration system was idle and between two chamber measurements. Similarly to the SRS, one chamber was measured for 3 minutes. After each chamber the isotopic signal of the reference air was measured for 3 minutes. The procedure gave a sequence of reference and analysis (soil CO_2_ efflux as sampled from the chamber) air for 3–3 minutes in one hour of operation.

### Data processing and modelling

Data processing and statistical analysis were done in R [46]. Gaussian error propagation was used to calculate propagated uncertainties for the averages and model parameters.

Diel patterns of respiration for R_s_, R_basal+myc_, R_basal_, R_myc+rhizo_, R_myc_ and R_rhizo_ were modeled using a sine wave function [13]:

$$R=y0+a\times sine\left[(\frac{2\times \pi \times TOD}{2400})+c\right]$$ (4)

where *y0* represents the mean respiration rate over the time period modeled (μmol CO_2_ m^-2^ s^-1^), *a* is diel amplitude (μmol CO_2_ m^-2^ s^-1^), *c* corresponds to the shift of minimum and maximum diel peaks (radians), and *TOD* is time of day in hundreds. Using parameter *c* we calculated the peak timing of respiration (PTR).

The goodness of model fit was quantified by the Nash–Sutcliffe model efficiency (NSE) coefficient, which is calculated similarly to the coefficient of determination, but ranges from -∞, indicating a better prediction of the observed values by the mean than by the model to 1, which points to a perfect match of the observed and modelled data.

We assumed that diel changes of R_basal_ were mainly driven by the temperature, since this plot had no connection with living plants. We also assumed that the diel pattern of R_myc+rhizo_, R_myc_ and R_rhizo_ was governed by the carbon allocation, because the temperature response was removed by the subtraction of R_basal_. Therefore, we used some parameters obtained from fitting Eq 4 on the component’s respiration pattern in order to estimate the temperature and carbon allocation driven part of soil respiration.

The following additive model (Eq 5) was a conceptual framework for the estimation of the actual share of the temperature driven response and of the potential diel course of the carbon allocation driven part of soil respiration.

Sine wave models were combined into an additive model in order to estimate the effects of temperature and carbon allocation on the respiration, as follows:

$$R_{s}=\left\{y1+a_{Rbasal}\times sine[(\frac{2\times \pi \times TOD}{2400})+c_{Rbasal}]\right\}+\left\{(1-y1)+a1\times sine[(\frac{2\times \pi \times TOD}{2400})+c_{Rmyc+rhizo}\}\right]$$ (5)

where *a*_*Rbasal*_ is the amplitude (a, μmol CO_2_ m^-2^ s^-1^) and *c*_*Rbasal*_ is the diel peak (c, radians) from the fitted Eq 4 on R_basal_ dataset, *y1* is the mean temperature driven part of soil respiration (μmol CO_2_ m^-2^ s^-1^), *c*_*Rmyc+rhizo*_ is the diel peak (radians) from the fitted Eq 4 on R_myc+rhizo_ dataset and *a1* is the amplitude of the carbon allocation driven part of R_s_ (μmol CO_2_ m^-2^ s^-1^) in the corresponding phenological period. In the first term of Eq 5 we used two model parameters of Eq 4 fitted on R_basal_ (*a* and *c*) for estimating the temperature response within soil respiration, while parameter *y1* was allowed to vary. In the second term of Eq 5 we estimated the carbon allocation driven part of soil respiration, only the diel peak of R_myc+rhizo_ (*c*_*Rmyc+rhizo*_) was kept constant with *a1* being allowed to vary.

We also used GPP data and the calculated components of soil respiration to calculate Pearson correlation coefficients at different time lags using ccf function (cross-correlation) of R.

During the isotopic measurements the reference and chamber air were measured sequentially, therefore reference values during chamber measurements were estimated by linear interpolation between the neighboring reference sequences.

δ^13^C values of the soil CO_2_ efflux were calculated using the isotopic mass balance approach in each plot:

$$\delta^{13}C_{R}=\frac{\delta^{13}C_{out}\times c_{out}-\delta^{13}C_{in}\times c_{in}\;}{c_{out}-c_{in}}$$ (6)

where δ^13^C_out_ and δ^13^C_in_ are the isotopic signature of the outgoing and incoming air of the chamber and c_out_ and c_in_ are the CO_2_ concentration of the of the outgoing and incoming air of the chamber, respectively.

$$\delta^{13}C=\frac{R_{sample}}{R_{standard}}-1$$ (7)

and *R* stands for the ^13^C:^12^C isotope ratio of the sample and the international VPDB standard (0.011182), respectively.

For the details of data processing of isotopic measurements see Balogh et al. [31].

## Results

### Meteorological conditions, CO_2_ uptake and respiration rates among years and phenological periods

**Fig 1:**
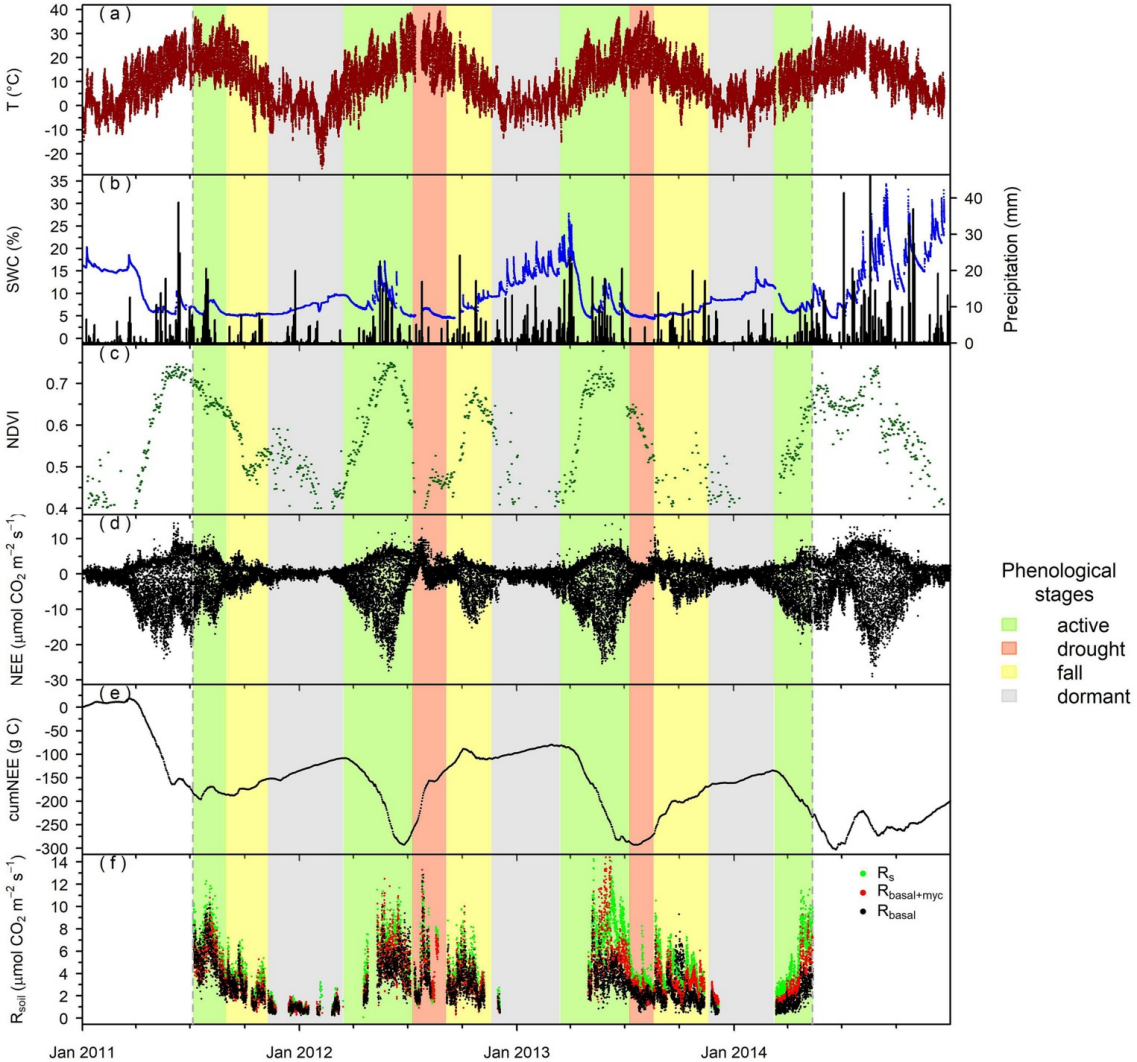
(a) Half-hourly average of air temperature (T, dark red dots), (b) soil water content (SWC, blue dots) at a depth of 5 cm and sum of precipitation (black bars), (c) normalized differential vegetation index (NDVI, green dots), (d) half-hourly net ecosystem exchange (NEE, black dots), (e) cumulative NEE (cumNEE, black line), and (f) hourly averages of R_s_, R_basal+myc_ and R_basal_ (green, red and black dots, respectively) during the study period in 2011–2014, at Bugac site. Phenological stages are shown by background colors (pale green as active, pale red as drought, yellow as fall and grey as dormant period).

The annual sums of precipitation in 2011 and 2012 were lower (436 and 431 mm year^-1^, respectively) than the ten–year average (575 mm), while it was close to that in 2013 (590 mm) and higher in 2014 (806 mm). Despite the low sum of precipitation in 2011 the grassland acted as a sink of carbon and no drought period could be distinguished within this year (Fig 1). The good water availability during the summer period in 2011 was due to the large amount of precipitation in 2010 (annual sum: 961 mm) and to the fact that 70%of the precipitation fell from March to September. This variability in water availability resulted in an overall sink activity in 2011 (-135 g C m^-2^ year^-1^), source activity in 2012 (38 g C m^-2^ year^-1^) and weak sink activity in 2013 and 2014 (-64 and -35 g C m^-2^ year^-1^, respectively).

We distinguished 4 phenological stages (active, drought, fall and dormant) within each year of the study period according to the net ecosystem exchange (NEE), air temperature (T), soil water content (SWC) and normalized difference vegetation index (NDVI) variations (Fig 1). The active periods were characterized by strong sink activity, while the drought periods showed strong source activity together with decreasing NDVI, high T and low SWC. The ecosystem showed a second or fall active period after the summer but this period was marked by weak source activity according to the cumulative NEE due to the decreasing temperature and NDVI. The dormant period was characterized by low temperatures, low NDVI and weak source activity (Fig 1, Table 2).

**Table 2:** Average soil CO_2_ effluxes (R_s_, R_basal+myc_ and R_basal_), air temperature (T), soil moisture (SWC) and net ecosystem exchange (NEE) for the phenological stages of the whole study period. Standard deviations of CO_2_ effluxes are shown (±SD).

*Phenological stage*	*R*_*s*_ *(μmol CO*_*2*_ *m*^*-2*^ *s*^*-1*^*)*	*R*_*basal+myc*_ *(μmol CO*_*2*_ *m*^*-2*^ *s*^*-1*^*)*	*R*_*basal*_ *(μmol CO*_*2*_ *m*^*-2*^ *s*^*-1*^*)*	*T (°C)*	*SWC* *(%)*	*NEE* *(μmol CO*_*2*_ *m*^*-2*^ *s*^*-1*^*)*
*active*	6.25 (±1.65)	5.01 (±1.8)	3.78 (±0.98)	15.14	8.7	-1.27
*drought*	4.00 (±0.99)	3.19 (±0.85)	2.81 (±0.72)	22.39	5.1	1.44
*fall*	3.93 (±1.04)	3.03 (±0.87)	2.63 (±1.17)	12.23	7.8	0.64
*dormant*	1.19 (±0.38)	1.11 (±0.25)	0.90 (±0.18)	1.30	12.1	0.29

Both R_s_ and R_basal+myc_ decreased by 36% on average in response to drought, while R_basal_ was less responsive and decreased by 26%. Average R_s_ and R_basal+myc_ were higher by 65% and 33% than R_basal_ in the active period and by 43% and 14% in the drought period, respectively. Average R_s_ and R_basal+myc_ were also higher than R_basal_ in the fall and dormant periods by 49%, 15% and by 31%, 23%, respectively (Table 2).

### Diel patterns of soil CO_2_ effluxes among the phenological stages

**Fig 2:**
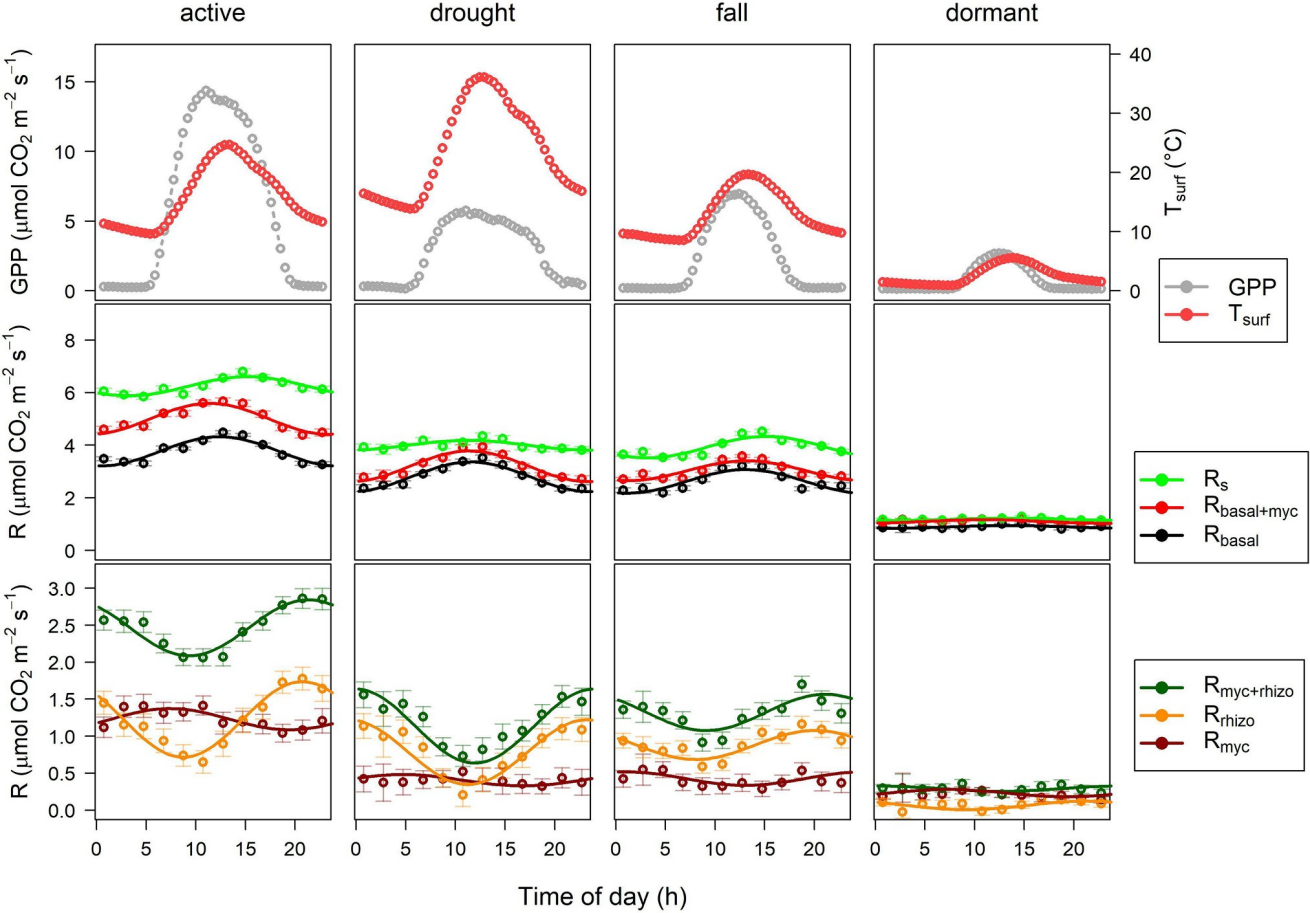
Diel patterns of GPP and T_surf_, (grey and red dots, respectively, upper panel), R_s_, R_basal+myc_ and R_basal_ (green, red and black dots with error bars, respectively, middle panel) and R_myc_, R_myc+rhizo_ and R_rhizo_ (dark red, dark green and orange dots with error bars, y = 0 represents R_basal_, lower panel) for the phenological stages during the whole study period at Bugac site. Lines represent fitted sine wave models (Eq 4) in middle and lower panels.

Since the SRS measured soil CO_2_ effluxes twelve times a day, we evaluated the diel changes in soil CO_2_ effluxes by binning the data by time of day (12 bins) for each phenological period and fitted Eq 4 on the datasets. Binned averages and fitted models are shown in Fig 2. The parameters of the sine wave function differed for R_s_, R_basal+myc_ and R_basal_ (Fig 2, middle panel). Peak R_basal_ occurred at about 12:00–14:00 hours (local time) at all phenological stages, while peak R_basal+myc_ occurred earlier in the active, drought and dormant periods (11:00–11.30). Peak R_s_ was observed much later than peak R_basal_ in the active period (15:30). During drought R_s_ peaked 4 hours earlier than in the active period and both peak R_s_ and R_basal+myc_ (both at 11:30) preceded peak R_basal_. Contrary to expectations, the largest amplitude (*a*) of R_s_ was found in the fall period rather than in the active period. The amplitude of R_basal+myc_ and R_basal_ were greater than the amplitude of R_s_ in the active and drought periods.

GPP was the highest at 11:15 in the active and drought periods but this peak shifted to 12:45 in the fall and dormant periods (Fig 2, upper panel). Peak R_basal_ was roughly coincident with the surface temperature (T_surf_) peaking within the same hour (Fig 2. middle panel), except in the active period when peak R_basal_ preceded the highest temperatures by more than 1 hour.

**Fig 3:**
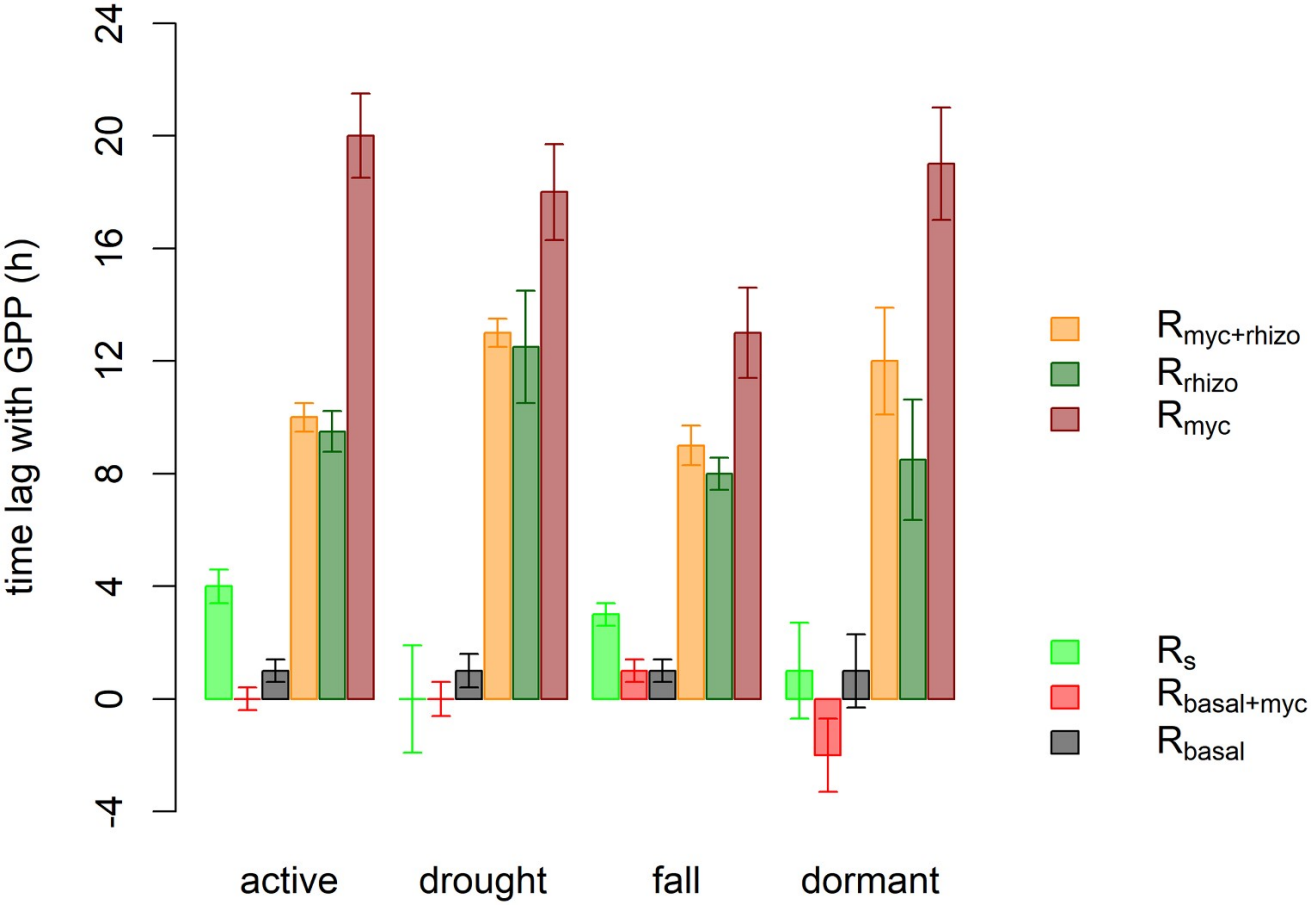
Average time lag between GPP peak and respiration peaks (Eq 4) in the different phenological stages. y = 0 line represents GPP peak, negative values mean that respiration preceded GPP.

R_myc+rhizo_, R_myc_, and R_rhizo_ showed much longer time lags with GPP (Fig 3). Peak R_myc+rhizo_ occurred in the evening and at night at 21:30–1:00 by 9–12 hours after GPP peak (11:30–12:45), while R_myc_ peaked at 1:30–7.30 by 13–20 hours after GPP. R_rhizo_ peaked earlier than R_myc+rhizo_ by about 0.5–1 hour (Fig 3, Table 3).

**Table 3:** Number of plot average values included in modelling (N), diel sine wave model parameters with propagated uncertainties (*y0*, *a*, *c* from Eq 4 (±SE)), peak timing of respiration (PTR) and Nash–Sutcliffe coefficient (NSE) for basal soil respiration component (R_basal_), mycorrhizal fungi and rhizospheric component (R_myc+rhizo_), mycorrhizal fungi component (R_myc_) and rhizospheric component (R_rhizo_) at phenological periods. Significance level of the parameters were P<0.001 or P<0.05 (*).

*Phenol*. *stage*	*plot*	*N*	*y0* *(μmol CO*_*2*_ *m*^*-2*^ *s*^*-1*^*)*	*a* *(μmol CO*_*2*_ *m*^*-2*^ *s*^*-1*^*)*	*c* *(radians)*	*PTR* *(hour*:*min)*	*NSE*
*active*	R_basal_	2867	3.77 (±0.04)	0.56 (±0.05)	4.58 (±0.09)	12:30	0.043
	R_myc+rhizo_	2861	2.47 (±0.05)	0.38 (±0.06)	2.23 (±0.13)	21:30	0.020
	R_myc_	2846	1.23 (±0.05)	0.14 (±0.06)*	5.89 (±0.4)	7:30	0.003
	R_rhizo_	2922	1.23 (±0.04)	0.51 (±0.05)	2.36 (±0.1)	21:00	0.033
*drought*	R_basal_	781	2.80 (±0.07)	0.56 (±0.09)	4.71 (±0.16)	12:00	0.050
	R_myc+rhizo_	777	1.14 (±0.06)	0.5 (±0.07)	1.57 (±0.13)	0:00	0.102
	R_myc_	747	0.40 (±0.05)	0.08 (±0.05)*	0.26 (±0.44)	5:00	0.007
	R_rhizo_	904	0.79 (±0.03)	0.44 (±0.04)	1.7 (±0.1)	23:30	0.118
*fall*	R_basal_	2304	2.63 (±0.04)	0.45 (±0.05)	4.31 (±0.09)	13:30	0.060
	R_myc+rhizo_	2293	1.32 (±0.04)	0.24 (±0.05)	2.23 (±0.18)	21:30	0.016
	R_myc_	2273	0.43 (±0.04)	0.09 (±0.05)*	1.18 (±0.41)	1:30	0.030
	R_rhizo_	2395	0.88 (±0.02)	0.2 (±0.03)	2.49 (±0.13)	23:30	0.023
*dormant*	R_basal_	777	0.90 (±0.01)	0.05 (±0.02)*	4.19 (±0.38)	14:00	0.008
	R_myc+rhizo_	776	0.29 (±0.02)	0.04 (±0.03)*	1.31 (±0.48)	1:00	0.005
	R_myc_	774	0.23 (±0.02)	0.05 (±0.02)*	5.89 (±0.52)	7:30	0.007
	R_rhizo_	903	0.07 (±0.01)	0.06 (±0.02)*	2.23 (±0.34)	21:30	0.010

Cross-correlation results showed similar time lags with GPP. The time lag at the maximum correlation between R_myc+rhizo_ and GPP was 5.5–11 hours, while a longer time lag (15–22 hours) was found between R_myc_ and GPP. The highest correlation coefficients were found in the active period at the time lags of 7.5 hours (r = 0.27, P<0.001) and 21.5 hours (r = 0.13, P<0.001) for R_myc+rhizo_ and for R_myc_, respectively.

The average diel range of respiration (2×*a*) compared to the average respiration rate (*y0*) was the highest in R_basal_ plot (40% in drought period), while it varied between 8% (dormant) and 21% (fall) in R_s_ plot (Table 3, S1 Table).

Fitting procedures of Eq 5 were successful for the active and fall periods only since *y1* parameter was not significant for the drought and dormant periods. We present here the results as a conceptual framework. The ratio of temperature and carbon allocation driven part of respiration was 0.55:0.45 in the active period, while 0.71:0.29 in fall (for the fitting results see supporting information S2–S5 Figs).

### Diel pattern of δ^13^C values of soil respiration in the phenological stages

**Fig 4:**
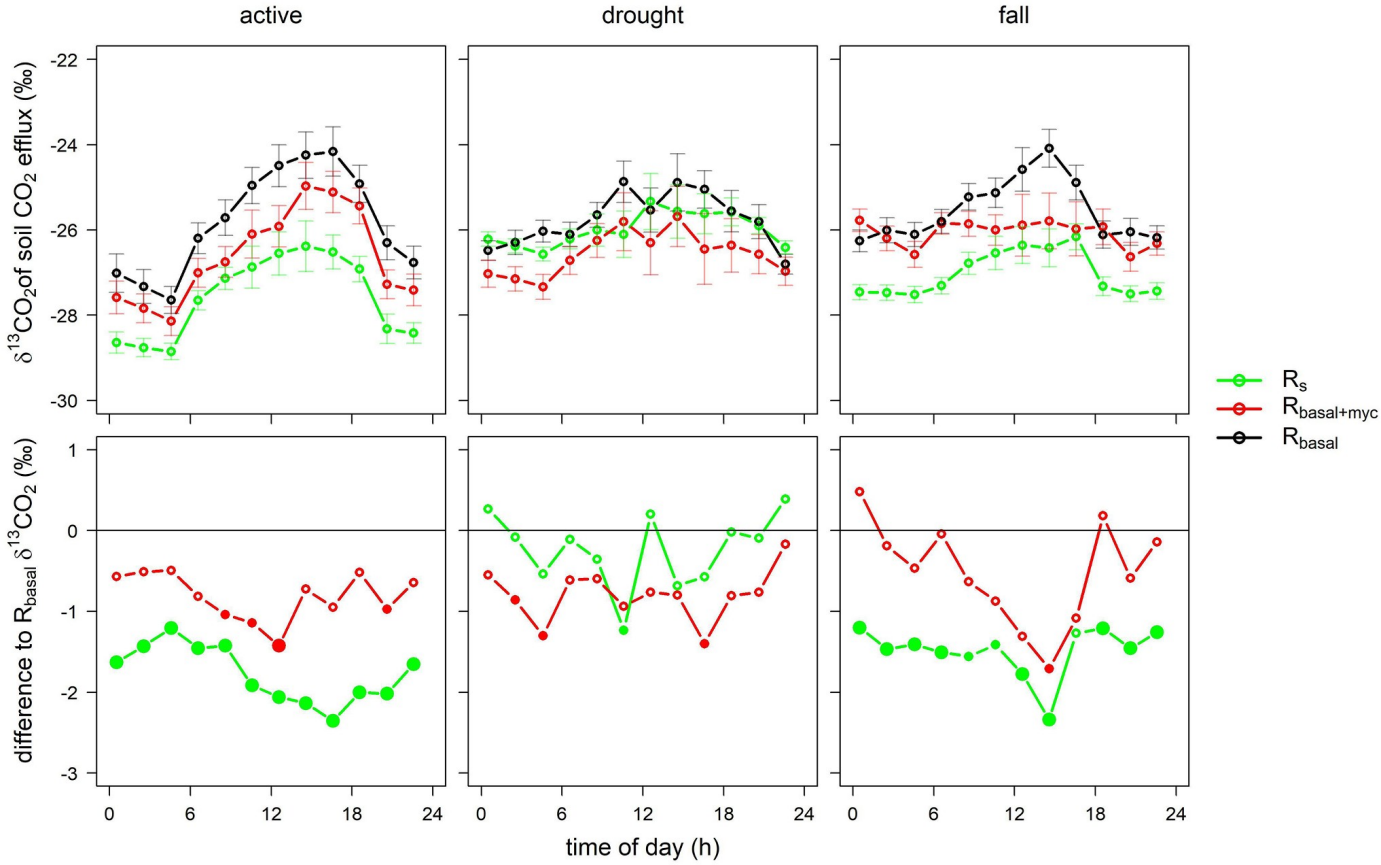
Diel pattern of δ^13^C of soil CO_2_ effluxes (green, red and black dots with error bars, upper panel) and the difference between δ^13^C_Rs_ and δ^13^C_Rbasal_ (green) and between δ^13^C_Rbasal+myc_ and δ^13^C_Rbasal_ (red, y = 0 represents δ^13^C_Rbasal_, lower panel). Significant differences from δ^13^C_Rbasal_ are shown by larger (P<0.05) and smaller (P<0.1) bold points.

Isotopic (^13^C) measurements were conducted in 2013 at three phenological stages: active (from 15^th^ May to 10^th^ July), a drought period from 10^th^ July to 19^th^ August, and a fall period from 20^th^ August to 11^th^ November. Average δ^13^C_Rs_ values were -27.6‰ (±1.2‰), -26.0‰ (±1.4‰), -27.0‰ (±1.0‰) (mean±SE), while average δ^13^C_Rbasal+myc_ values were -26.6‰ (±1.5‰), -26.6‰ (±1.9‰), -26.1‰ (±1.5‰) and δ^13^C_Rbasal_ values were -25.8‰ (±1.5‰), -25.8‰ (±1.4‰), -25.5‰ (±1.2‰) in the active, drought and fall periods, respectively. δ^13^C data were also grouped and averaged by time of day for each phenological period (Fig 4, upper panel).

Diel pattern of δ^13^C of all soil CO_2_ effluxes was the most pronounced in the active period in all of the measured plots, and δ^13^C of respiration was more depleted during nighttime than during daytime at all phenological stages (Fig 4 upper panel). δ^13^C values of the reference air (chamber inlet) ranged between −16 and −8‰ and showed similar patterns with nights being more depleted than daytime periods.

**Fig 5:**
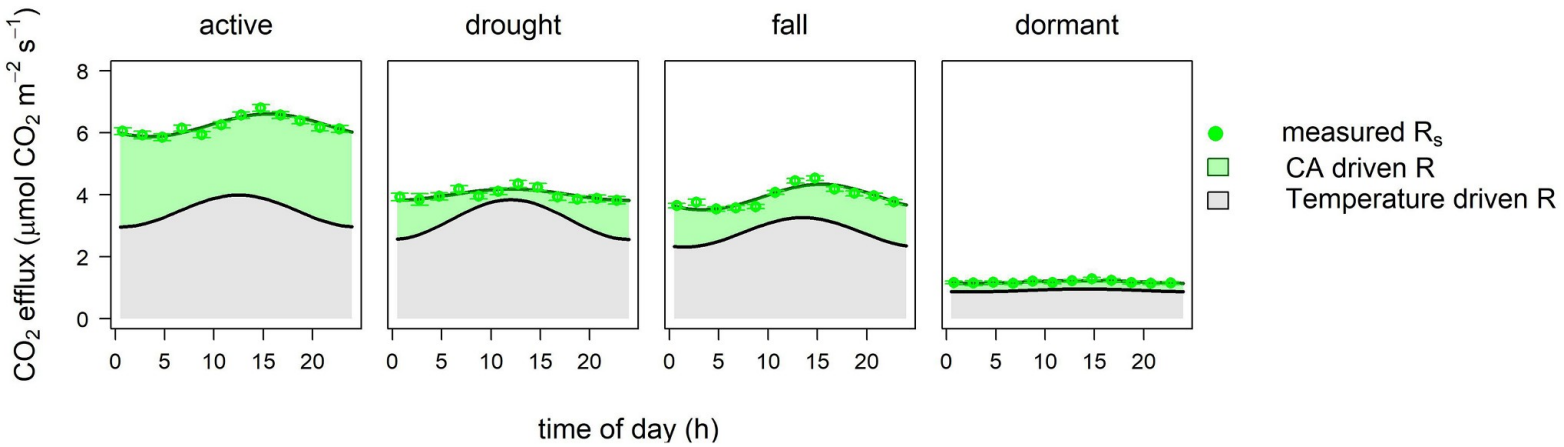
Conceptual model of temperature and carbon allocation driven parts of soil respiration based on Eq 5. The share of temperature driven respiration (gray area) and carbon allocation driven rhizospheric respiration (green area), as well as measured R_s_ averages with standard errors (green dots with error bars) are shown.

The largest differences between δ^13^C_Rs_ and δ^13^C_Rbasal_ and between δ^13^C_Rbasal+myc_ and δ^13^C_Rbasal_ were also found in the active period and the diel course of these differences showed similar pattern to R_myc+rhizo_ and R_myc_ during daytime (cf. Figs 2 and 5). The highest deviation of δ^13^C_Rs_ from δ^13^C_Rbasal_ was observed in the afternoon and evening (12:30–20:30), while the lowest deviation was observed in early morning (2:30–8:30). During the drought period δ^13^C_Rs_ values were between δ^13^C_Rbasal_ and δ^13^C_Rbasal+myc_ and the diel changes of δ^13^C were not pronounced, while the patterns observed in the active period returned in fall. δ^13^C_Rs_ values were significantly different from δ^13^C_Rbasal_ in the active and fall periods, while δ^13^C_Rbasal+myc_ showed significant differences from δ^13^C_Rbasal_ in only a few cases (Fig 4, lower panel).

## Discussion

The disturbance of the soil structure in mesh-collar (root/mycorrhiza exclusion) studies is an inevitable part of the procedure and necessitates the exclusion of data of the initial period from evaluation. In order to avoid the artifacts due to the installation procedure the measurements did not start until 10 months following the installation of the tubes. At the end of the measurements we found higher hyphal density in R_basal+myc_ plots than in R_s_ plot [39] and the absence of roots in R_basal_ and R_basal+myc_ plots was also verified. Moreover, the lack of roots resulted in higher soil moisture in R_basal+myc_ and R_basal_ plots than in R_s_ plot [39]. These differences could cause the overestimation of R_myc_ values, while R_basal_ was probably underestimated due to the exclusion of fresh SOM supply (no dead roots in the plot) and the lack of rhizodeposits accelerating SOM decomposition [33]. Soil temperature at a depth of 5 cm was also higher in R_basal_ plots than in R_s_ plots. Due to these differences between the plots it was not possible to use R_basal+myc_ and R_basal_ respiration rates as direct estimations of heterotrophic and mycorrhizal fungi respiration. Instead, we used the calculated R_myc+rhizo_, R_rhizo_ and R_myc_ rates as references to study the diel pattern of the mycorrhizal fungi and rhizospheric components of soil respiration. Since R_basal_ had no connection with the plants we could assume that the diel changes of R_basal_ were driven by the temperature. By subtracting R_basal_ from R_s_ we removed this temperature effect and could use R_myc+rhizo_ to study the effect of carbon allocation.

### The effect of carbon allocation on diel pattern of R_s_

The time lag between R_s_ and its drivers was studied in different ecosystems and on multiple time scales [18,27]. Some studies found that the rate of photosynthesis had a stronger effect on root respiration than changes in temperature did [19,27,47]. Based on the daily averages of GPP and R_s_ we found 0 day lag between them in the most active period (May-July) indicating that the time lag was shorter than one day [39]. Labelling studies using stable isotopes of carbon also found short-term (<1 day) coupling between photosynthetic uptake of CO_2_ and soil respiration [17,48]. However, temporal changes in photosynthetic substrate supply to the rhizosphere may also be apparent in heterotrophic respiration after a time lag [6,14]. Tracer amount peaked 24 hours after pulse-labelling in decomposer fungi and bacteria in a study by De Deyn et al.[49]. Therefore, the observed time lag between GPP and R_myc+rhizo_ included the response of the whole mycorrhizosphere. In this study R_basal+myc_ always peaked earlier than R_basal_ in all phenological periods suggesting that mycorrhizal fungi respiration also had a diel pattern, which is not temperature dependent. By subtracting R_basal_ from R_basal+myc_ we were able to remove the temperature effect from the diel pattern, allowing us to observe the single effect of carbon allocation on R_myc_. This component peaked in the morning 13–20 hours after GPP peak. Similarly, the diel pattern of R_rhizo_ could also show this effect in the rhizosphere. R_rhizo_ peak preceded R_myc+rhizo_ peak by 0.5–3.5 hours and R_myc_ peak by 5.5–10.5 hours in all phenological periods but it must be noted that R_rhizo_ included the respiration of rhizospheric organisms as well and not just that of the roots. Therefore, root respiration might have even shorter time lag with GPP than R_rhizo_, i.e. less than 8–12 hours (Fig 3).

Based on the observed order of component peaks we can assume that the newly assimilated carbon induced a propagating pattern of root—rhizosphere microorganisms—mycorrhizal fungi respiration within 24 hours. The magnitude of this effect could be apparent in the amplitude of the fitted models (e.g. amplitude of R_myc+rhizo_), while the remaining part of the respiration could be fueled by stored carbohydrates. Root-stored photosynthates can buffer the changes in respiration during the decline in the supply of photosynthates not only in the long run [50] but on diel scale as well [6]. Transitory starch also plays an important role as substrate for nocturnal respiration balancing the substrate supply of the rhizosphere [48]. Moreover, considerable rhizospheric priming effect could be presented only in R_s_ plot containing roots since root exudates are the main fuel of the process and can act in the vicinity of roots [33]. Besides the effect of carbon allocation, the observed diel pattern of R_s_ could partly be attributed to the link between water transport and CO_2_ flux within the plants. It was recently found that CO_2_ transported in the transpiration stream could reduce apparent root respiration [51,52], i.e. CO_2_ production in the soil [37]. We hypothesize that all of these effects could result in the relatively low diel variability of R_s_. Average diel range of respiration (2×*a*, Eq 4) compared to the average respiration rate (*y0*, Eq 4) was higher in R_basal_ plot (40% in drought period) than in R_s_ plot, which varied between 8% (dormant) and 21% (fall). Diel range of R_s_ was small even under drought conditions (10%).

### Effects of drought on diel patterns of R_s_

All types of measured soil CO_2_ effluxes (R_s_, R_basal+myc_, R_basal_) decreased under dry conditions but the biggest decline was observed in soil respiration (R_s_). Only this plot included all of the autotrophic components thus this strong response could be attributed to the autotrophic part of soil respiration. This finding is in line with previous studies where reduced autotrophic respiration was found in grassland ecosystems as a response to drought [19,48] due to the reduced assimilate supply and to the changes in allocation strategy [17]. The change in R_myc_ also supports this finding since it had much higher share in R_basal+myc_ in the active period than during the drought suggesting a disproportionate reduction of carbon allocation into roots and root-associated organisms. Carbon transfer to mycorrhizal fungi could vary several-fold seasonally [16] and drought can induce increased allocation of carbon to root storage at the expense of root respiration [17,48]. Increased time lag between GPP peak and R_myc+rhizo_ under drought (+2.5 hours as compared to the active period) can also be explained by this process and by the reduced speed of carbon allocation [17].

Contrary to our expectations, the amplitude of R_s_ was small in the drought period as compared to the amplitude of R_basal_ resulting in large diel amplitude of R_myc+rhizo_ (44% of mean R_myc+rhizo_). This phenomenon could only partly be attributed to the changes in the photosynthetic supply due to the small GPP in this period. Although the amount of transitory starch declines, the increased contribution of storage pools in root respiration [6,53] could reduce this amplitude. However, the effect of another factor could also modify this diel pattern. While average diel changes of soil moisture were not significant, even a small decrease in the level of moisture in the rhizosphere could cause a decline in CO_2_ efflux under water shortage. Daytime moisture depletion of the rhizosphere due to evapotranspiration could significantly affect respiration rates with rapid decline of R_s_ being found under 6% SWC in this ecosystem in our former studies [39,5]. Moreover, most CO_2_ production takes place close to the surface (0–8 cm) in this ecosystem [37] and this upper layer is the most exposed to the daytime drying—nighttime rewetting (dew formation, water redistribution) cycles.

### Diel changes in isotopic signal (δ^13^C) of soil respired CO_2_

A C4 grass (*Cynodon dactylon*) was also present in the study site potentially modifying the δ^13^C of the respired CO_2_. Its cover was about 10% in the pasture [35], but it was less frequent (i.e. less than 5%) in the experimental area. Modelling results showed a strong decline in the autotrophic components in response to drought [31,39], although the observed shift (increase) in δ^13^C_Rs_ during drought was also coincident with the maximum abundance of this species in July-August.

Uncertainties related to field measurements of isotopic signals of soil CO_2_ efflux were also reported [54,55,56]. The observed diel pattern of δ^13^C of respiration (more depleted during nights) can be explained by the effect of the non-steady-state conditions in the soil profile due to the nighttime increase of CO_2_ concentration over the surface [57]. Drying of the surface layers can also modify δ^13^CO_2_ since heterotrophic respiration could be restricted to the deeper layers of the soil [56]. These effects can be considerable at the study site where still conditions often occur during nighttime and dry conditions are also frequent in the vegetation period [47]. However, we can assume that all plots (R_s_, R_basal+myc_, R_basal_) were affected similarly by these conditions, therefore the observed diel changes in the difference between δ^13^C_Rs_ and δ^13^CR_basal_ and in the difference between δ^13^C_Rbasal+myc_ and δ^13^CR_basal_ could be governed by the diel changes in the contribution made by rhizospheric and mycorrhizal fungi to R_s_. These changes in the isotopic signals were most pronounced in the active period. The largest deviation of δ^13^C_Rs_ from δ^13^CR_basal_ was observed in the afternoon, following the peak in GPP by a few hours delay (~4 hours), while it was the closest to δ^13^CR_basal_ at early morning. The largest deviation of δ^13^C_Rbasal+myc_ from δ^13^CR_basal_ was observed in the morning, roughly coincident with the GPP peak (-2-0 hours). Beside the changes in the component’s contribution to soil respiration, post-carboxylation fractionation could also cause changes in the isotopic signal of the rhizospheric and mycorrhizal fungi components [57]. Phloem sugars can be ^13^C enriched during night-time, while ^13^C depleted during day-time due to the isotope fractionation of enzymatic reactions [58]. Thus, our observations in terms of the diel pattern of isotopic signals also support that the carbon uptake and root respiration have short-term (few hours) coupling, i.e. carbon allocation is fast to the roots, while it takes more time (~20–24 hours) to reach and affect the respiration of mycorrhizal fungi [49].

### The integrated effects of temperature and carbon allocation govern diel variability of R_s_

We used a combined sine wave model (Eq 5) to estimate the share of temperature-driven and carbon allocation driven parts of soil respiration (Fig 5). The presented conceptual model of the superposed responses could describe the causes behind the diel variability of the soil CO_2_ efflux. We assume that this short-term coupling of the two processes (carbon allocation and temperature effects) could be typical in grasslands, where the speed of the phloem transport and the short distance could induce changes in soil CO_2_ efflux on the same day [14,59].

According to our model, the temperature was the major factor affecting soil respiration on the diel time scale in every phenological stages. The effect of carbon allocation was most pronounced in the active period reaching 45% share in soil respiration.

Our model concept does not separate the autotrophic and the heterotrophic components of soil respiration. More importantly, however, it separates the temperature- and the carbon allocation driven parts of the respiration, with the temperature driven part including both the heterotrophic and autotrophic response. Similarly, the carbon allocation driven part includes some heterotrophic respiration (priming) due to the root exudation in the rhizosphere [34].

## Conclusions

Our results suggest that belowground carbon allocation can influence the diel pattern of soil respiration in grasslands. Based on the observed time lags between GPP and soil respiration components we can assume that the newly assimilated carbon induced a propagating pattern of root—rhizosphere microorganisms—mycorrhizal fungi respiration within 24 hours. Although temperature had the strongest effect on soil respiration according to our model, carbon allocation clearly modified the temperature driven pattern of soil respiration in the growing season. Therefore, even the time-lagged apparent temperature responses of soil respiration could contain significant respiration activities not driven by the temperature. Besides temperature and carbon allocation, soil moisture could also have an effect on the diel scale by the reduction of the autotrophic component during drought and also by the modification of the carbon allocation pattern.

## Supporting information

S1 Fig Schematic map of the study area. (JPG)

S2 Fig Normalized diel pattern of measured R_s_ values in the active period, modelled temperature driven respiration, modelled carbon allocation driven respiration and modelled soil respiration. (JPEG)

S3 Fig Normalized diel pattern of measured R_s_ values in the drought period, modelled temperature driven respiration, modelled carbon allocation driven respiration and modelled soil respiration. (JPEG)

S4 Fig Normalized diel pattern of measured R_s_ values in the fall period, modelled temperature driven respiration, modelled carbon allocation driven respiration and modelled soil respiration. (JPEG)

S5 Fig Normalized diel pattern of measured R_s_ values in the dormant period, modelled temperature driven respiration, modelled carbon allocation driven respiration and modelled soil respiration. (JPEG)

S1 Table Number of plot average values included in modelling (N), diel sine wave model parameters with propagated uncertainties (*y0*, *a*, *c* from Eq 4 (±SE)), peak timing of respiration (PTR) and Nash–Sutcliffe coefficient (NSE) for R_s_ and R_het+myc_, at phenological periods. (DOCX)

S1 File Soil CO_2_ effluxes of the measured plots and eddy-covariance derived GPP dataset, 2011–2014, Bugac site. (CSV)

S2 File Isotopic signals of the measured CO_2_ effluxes, 2013, Bugac site. (CSV)
