# Supplementary figures and images for: Separating the effects of temperature and carbon allocation on the diel pattern of soil respiration in the different phenological stages in dry grasslands
Source: PLoS One. 2019 Oct 17;14(10):e0223247. doi: 10.1371/journal.pone.0223247 (PMC6797092; doi:10.1371/journal.pone.0223247)

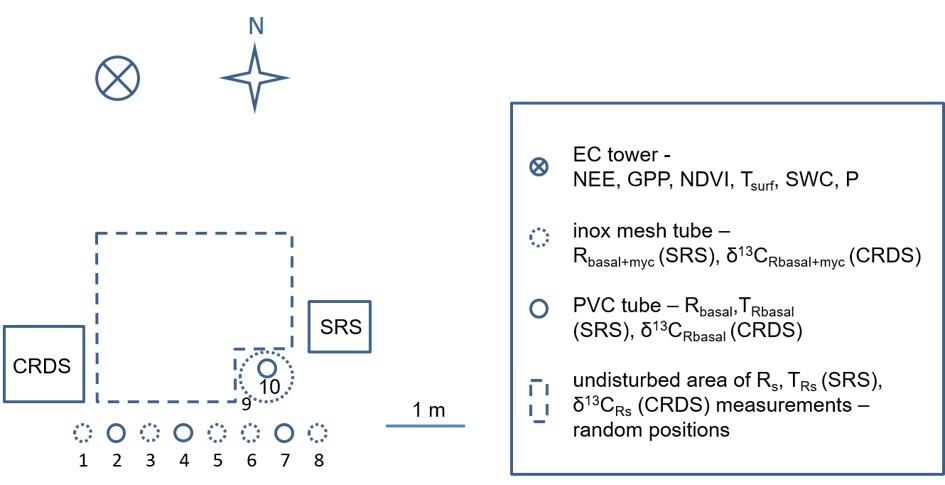

Supplement: S1 Fig — (JPG) [file pone.0223247.s001.jpg]

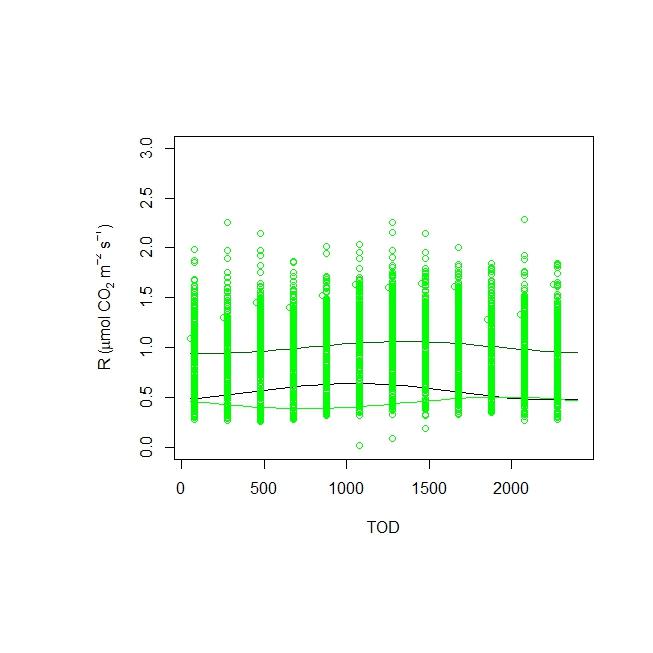

Supplement: S2 Fig — (JPEG) [file pone.0223247.s002.jpeg]

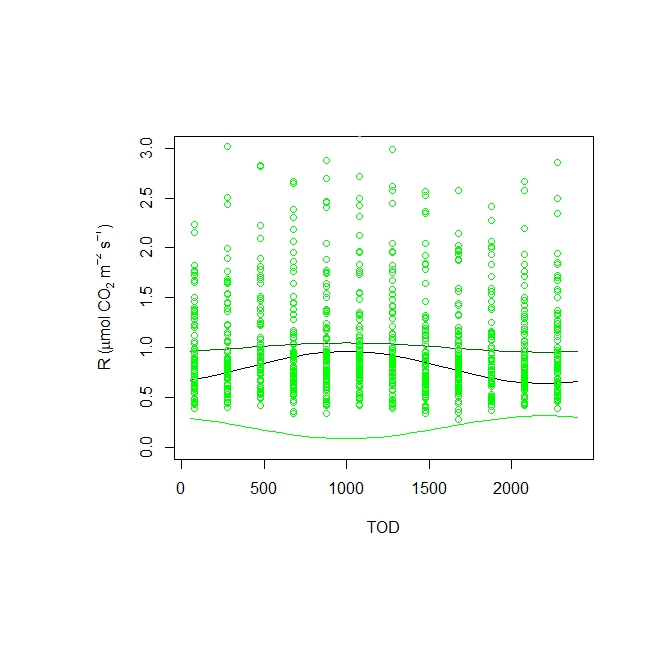

Supplement: S3 Fig — (JPEG) [file pone.0223247.s003.jpeg]

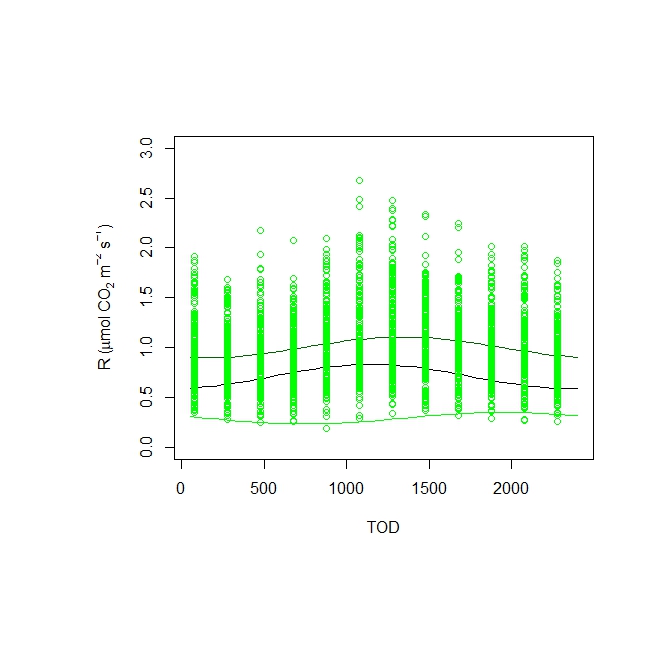

Supplement: S4 Fig — (JPEG) [file pone.0223247.s004.jpeg]

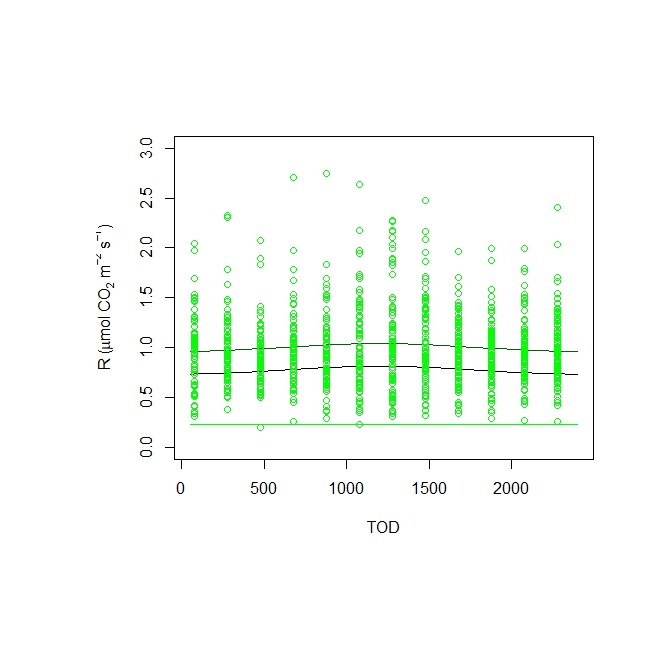

Supplement: S5 Fig — (JPEG) [file pone.0223247.s005.jpeg]
