# Supplementary material for: Separating the effects of temperature and carbon allocation on the diel pattern of soil respiration in the different phenological stages in dry grasslands
Source: PLoS One. 2019 Oct 17;14(10):e0223247. doi: 10.1371/journal.pone.0223247 (PMC6797092; doi:10.1371/journal.pone.0223247)
Supplement: S1 Table — (DOCX) [file pone.0223247.s006.docx]

| *Phenological*  *stage* | *plot* | *N* | *y0* | *a* | *c* | *PTR* | *NSE* |
| --- | --- | --- | --- | --- | --- | --- | --- |
| *active* | R_s_ | 2966 | 6.24 (±0.05) | 0.36 (±0.07) | 3.79 (±0.16) | 15:30 | 0.012 |
|  | R_het+myc_ | 2927 | 5.01 (±0.05) | 0.59 (±0.07) | 4.84 (±0.11) | 11:30 | 0.030 |
| *drought* | R_s_ | 950 | 4.00 (±0.08) | 0.18 (±0.1)^*^ | 4.84 (±0.5) | 11:30 | 0.004 |
|  | R_het+myc_ | 905 | 3.20 (±0.07) | 0.58 (±0.08) | 4.84 (±0.15) | 11:30 | 0.050 |
| *fall* | R_s_ | 2448 | 3.93 (±0.04) | 0.41 (±0.05) | 3.79 (±0.1) | 15:30 | 0.040 |
|  | R_het+myc_ | 2403 | 3.03 (±0.03) | 0.38 (±0.03) | 4.31 (±0.09) | 13:30 | 0.051 |
| *dormant* | R_s_ | 947 | 1.18 (±0.02) | 0.05 (±0.02)^*^ | 4.19 (±0.45) | 14:00 | 0.005 |
|  | R_het+myc_ | 909 | 1.11 (±0.02) | 0.07 (±0.02)^*^ | 4.97 (±0.35) | 11:00 | 0.009 |
